# Detection of Chloroalkanes by Surface-Enhanced Raman Spectroscopy in Microfluidic Chips

**DOI:** 10.3390/s18103212

**Published:** 2018-09-23

**Authors:** Zdeněk Pilát, Martin Kizovský, Jan Ježek, Stanislav Krátký, Jaroslav Sobota, Martin Šiler, Ota Samek, Tomáš Buryška, Pavel Vaňáček, Jiří Damborský, Zbyněk Prokop, Pavel Zemánek

**Affiliations:** 1 Institute of Scientific Instruments of the CAS, v.v.i., Czech Academy of Sciences, Kralovopolska 147, 61264 Brno, Czech Republic; martink@isibrno.cz (M.K.); jezek@isibrno.cz (J.J.); kratky@isibrno.cz (S.K.); sobota@isibrno.cz (J.S.); siler@isibrno.cz (M.S.); osamek@isibrno.cz (O.S.); pavlik@isibrno.cz (P.Z.); 2 Loschmidt Laboratories, Department of Experimental Biology and RECETOX, Faculty of Science, Masaryk University, Kamenice 5/A13, 62500 Brno, Czech Republic; bary@mail.muni.cz (T.B.); PavelVanacek@seznam.cz (P.V.); jiri@chemi.muni.cz (J.D.); zbynek@chemi.muni.cz (Z.P.)

**Keywords:** surface enhanced Raman spectroscopy, microfluidics, Klarite 312, chloroalkane, 1,2,3-trichloropropane

## Abstract

Optofluidics, a research discipline combining optics with microfluidics, currently aspires to revolutionize the analysis of biological and chemical samples, e.g., for medicine, pharmacology, or molecular biology. In order to detect low concentrations of analytes in water, we have developed an optofluidic device containing a nanostructured substrate for surface enhanced Raman spectroscopy (SERS). The geometry of the gold surface allows localized plasmon oscillations to give rise to the SERS effect, in which the Raman spectral lines are intensified by the interaction of the plasmonic field with the electrons in the molecular bonds. The SERS substrate was enclosed in a microfluidic system, which allowed transport and precise mixing of the analyzed fluids, while preventing contamination or abrasion of the highly sensitive substrate. To illustrate its practical use, we employed the device for quantitative detection of persistent environmental pollutant 1,2,3-trichloropropane in water in submillimolar concentrations. The developed sensor allows fast and simple quantification of halogenated compounds and it will contribute towards the environmental monitoring and enzymology experiments with engineered haloalkane dehalogenase enzymes.

## 1. Introduction 

Raman spectroscopy is an instrumental technique employing lasers for label free, fast, non-destructive and non-contact analysis of chemical compounds and their mixtures [1,2]. Probably the major drawback of Raman spectroscopy is its relatively low sensitivity. Only about one in 10^7^ photons [3] scattered from the sample arise from Raman-Smekal effect [4], aka Raman scattering [5]. This limits Raman spectroscopy in its capacity to detect trace amounts of analytes. The Raman signal intensity largely depends on the Raman cross-section of the molecule. This is proportional to its polarizability, which is in turn dictated by the chemical structure. Surface-enhanced Raman spectroscopy (SERS) [6], allows for amplification of the Raman signal by several orders of magnitude on nanostructured metal surfaces, so that it is in certain cases possible to observe signals even from individual molecules [7,8,9]. The enhancement is provided by the interaction of the localized plasmon oscillation with the electrons in the molecular orbitals of the studied molecule. The magnitude of the electromagnetic (EM) signal enhancement differs for various molecules between 10^8^ and 10^12^, but the average enhancement factors are in the order of 10^4^–10^6^ [10]. Different source puts the theoretical limit to the EM enhancement at 10^11^ [11]. In certain cases, an additional chemical signal enhancement was reported, reaching magnitude 10^2^–10^4^ [12,13]. Maximal total experimental averaged enhancement factors (EM + chemical enhancement) are on the order of 10^11^–10^12^ [14]. The main prerequisite for successful SERS detection is a close proximity of the molecule to the plasmonic structure, since the plasmonic field effectively reaches only a few nanometers far from the surface [15]. Several SERS detection techniques therefore require drying of the specimen on the substrate, in order to concentrate the analyte on the surface and to prevent its diffusion away from the substrate into the solution [16]. The diffusion-mediated effect of reduced SERS signal, observed when attempting SERS of dissolved molecules, could still be potentially intensified by the local heating of the substrate [17]. However, with volatile molecules such as 1,2,3-trichloropropane (TCP), drying of the sample might introduce serious errors. Additionally, such approach precludes the possibility to build a flow-through SERS sensor.

The emergence of several successive generations of improved, cheaper and smaller computers, optical sensors and lasers allowed for fast development in the field of optical (including ultraviolet (UV) and infrared (IR)) analytical methods, such as various forms of spectroscopy, including Raman and SERS. The new analytical solutions in this area frequently offer low price, portability, modularity, high sensitivity, easy sample preparation [18,19], and in medical diagnostics they follow the trend of point-of-care systems [9,20]. Microfluidic solutions for sample handling are also becoming more frequently incorporated into such products and sometimes play a crucial role in enhancing the throughput of the analytical systems, such as, e.g., with droplet microfluidics [21]. The availability of cheap and fast nanostructuring techniques such as pressure molding for microfluidic chip fabrication makes the incorporation of a plasmonic structure such as SERS substrate into the microfluidic chip an attractive option, even for mass fabrication.

A large variety of SERS substrates were reported to be effective for detection of minute amounts of pharmaceuticals, toxins and pollutants, as well as other biologically important molecules [20,22,23], including various microfluidic analytical SERS devices [24,25,26]. While the suspended nanoparticles can be made chemically more reactive towards certain chemicals, or they can be made in situ [27], the signal reproducibility is notoriously hard to obtain due to the stochastic nature of the created hotspots of plasmonic oscillations [28,29]. The surface structures for SERS, due to their periodic pattern, possess more uniform response, which is crucial for repeatable results.

Our SERS substrate named SK307 is based on a silicon chip, on which structures were fabricated using electron beam lithography and wet etching to achieve a pattern of inverted pyramids on the surface, which was then covered by gold layer of defined thickness and roughness. With SK307, we developed a simple and versatile optofluidic SERS-based diagnostic and analytical system which provides fast and reliable quantitative measurement of organic chemicals, such as halogenated hydrocarbons. Specifically we were able to detect a toxic environmental pollutant 1,2,3-trichloro-propane (TCP) [30,31,32] in water at submillimolar concentrations. We also tested our optofluidic SERS system with other analytes such 2,3-dichloropropan-1-ol (DCP) and trichloromethane (CHCl_3_). Additionally, we assessed the influence of the pH change and the NaCl concentration on the Raman signal and found improved conditions for TCP SERS measurement.

## 2. Materials and Methods

### 2.1. Fabrication of the SERS Substrate

**Figure 1:**
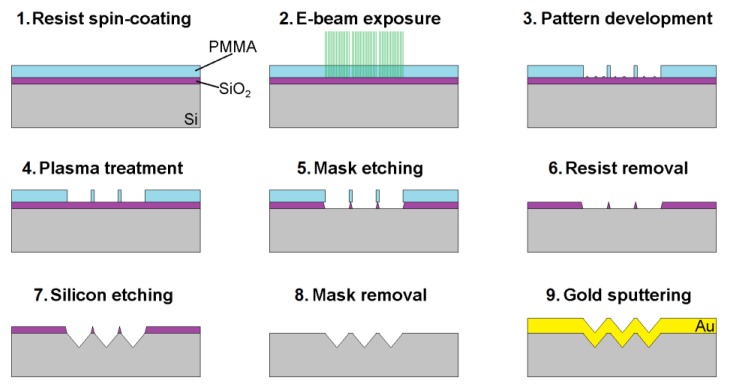
Schematic description of the process for SERS (Surface-enhanced Raman spectroscopy) substrate preparation. See detailed description of the process in the main text.

Silicon (100) was used as a substrate for the sample preparation. Silicon dioxide mask (thickness of 100 nm) was prepared by thermal oxidation. Subsequently, thin layer of PMMA with the thickness of 80 nm was spin coated on the oxidized substrate. An E-beam system Raith (Vistec, Jena, Germany) EBPG5000+ ES performed the exposure of the desired pattern. The pattern consisted of a simple square lattice with the pitch of 1325 nm (square with the side length of 900 nm was the exposed part) that covered the area of 4 × 4 mm^2^. Alcohol-free developer was used for the pattern development. A brief oxygen plasma treatment was applied to the whole substrate in order to remove the resist residue after the development step. The etching of silicon dioxide mask was carried out by buffered oxide etchant (BOE, a mixture of ammonium fluoride and hydrofluoric acid). After pattern transfer into the silicon dioxide mask, the resist layer was removed by oxygen plasma. Reverse pyramidal structures were etched into the silicon substrate by aqueous solution of potassium hydroxide (40% *w/w* at 60 °C). The rest of the silicon dioxide mask was removed by BOE after the silicon etching. Afterwards, the whole substrate was diced into small chips (10 × 10 mm^2^). The final step was the magnetron sputtering of 300 nm gold (Au) layer. The resulting pyramidal arrays had square base, 1325 nm pitch length and 900 nm pit depth. The whole manufacturing process is shown on Figure 1.

**Figure 2:**
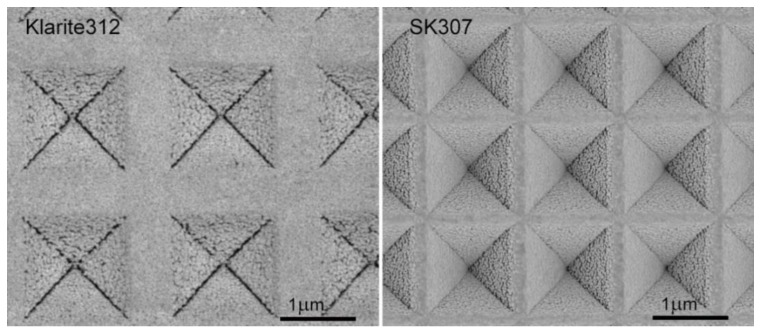
SEM images of the SERS substrates Klarite 312 and SK307. The images were obtained with scanning electron microscope Magellan (FEI, Hillsboro, OR, USA; TLD; 1–2 kV; 6.3–25 pA; 2.6–3.0 mm WD; 15,000–20,000× magnification). SK307 has about twice the number of pyramids per unit area than Klarite 312. This was achieved both with smaller pyramids and narrower gaps between them. The granularity of the Au surface is essential for SERS activity. The scale-bars are 1 μm.

Our design was inspired by the well-known commercial SERS substrate Klarite 312 (Renishaw Diagnostics, Glasgow, UK; production has been discontinued). Klarite 312 is based on inverted pyramids with 2000 nm pitch length and 1000 nm pit depth, has square cross section and is fabricated on a silicon substrate [33]. Figure 2 shows the detail of the prepared structure of SK307 compared with Klarite 312. The granular character of the Au surface is essential for the function of the SERS substrate. We reached maximal SERS signals of TCP with closely packed granules with average diameter about 60 nm and average height in the center about 20 nm (determined by AFM, Atomic force microscopy). The substrate was always manipulated with clean tweezers in laminar box. Care was taken to keep the substrate perfectly clean. The experiments were always conducted on fresh substrates, maximum time for single substrate to be continuously used was 90 min, after which the substrate was removed from the microfluidic chip and recycled by thorough cleaning and re-sputtering with gold.

### 2.2. Fabrication of the Microfluidic Chip

**Figure 3:**
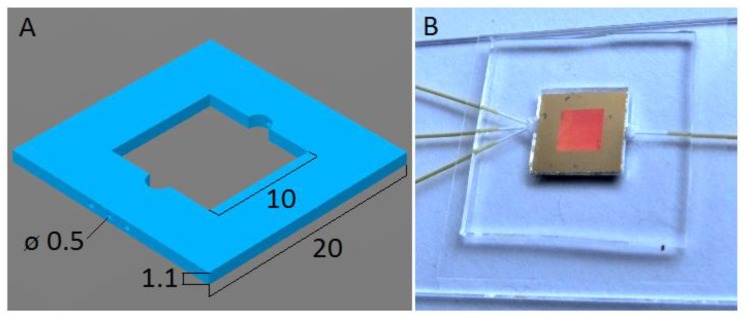
(**A**). Schematic image of the PDMS chip for microfluidic SERS. The chip is square shaped, with square substrate chamber in the center, with 3 inlets (left) and 1 outlet (right) for capillary tubes. Dimensions are in millimeters. (**B**). Photograph of the complete microfluidic chip with the SERS substrate in the microfluidic chamber on a microscope slide covered by a cover slip and the capillary tubing connected.

Our microfluidic chips were fabricated from poly(dimethyl)siloxane (PDMS) polymer and glass. The microfluidic chip used for SERS experiments with TCP was designed as a simple square frame (housing the SERS chip) with only three short and wide input ports (for sample, standard, and diluent) and one output, see Figure 3. The main reason for this design was to minimize the time the TCP solution spends in contact with PDMS, since TCP easily passes through PDMS, effectively decreasing its concentration in the solution. We used a mold fabricated from two-component resin Smooth-Cast 300 (Smooth-On, Inc., Macungie, PA, USA). To prepare the chamber for the SERS substrate, the resin was poured on a stub made from four thin rectangular sections of microscope glass slide glued on a flat glass tile to form a square cell (20 mm × 20 mm) with hollow center (10 mm × 10 mm), with thickness of 1.1 mm. After the resin hardened, the glass stub was removed and the holes for the microfluidic tube ports were drilled through the sides of the mold. The wires for tubing ports were placed into the holes and pushed all the way towards the central block that will form the chamber. Degassed PDMS mixture (Sylgard 184 by Dowsil/Dow Corning, Midland, MI, USA; base to curing agent ratio of 10:1) was then poured into the mold, which was immediately covered by a microscope slide, cured on a heated ceramic surface at 65°C for 4 hours and then demolded. 

### 2.3. Insertion of the Sers Substrate into the Microfluidic Chip and Completion of the Fabrication

The microfluidic chamber was prepared by gentle excision of PDMS overhangs with a sharp scalpel and subsequent transfer of the PDMS part on a clean and dry microscope slide. The fresh SERS substrate is transferred by tweezers and gently pushed into the tight PDMS frame. After the SERS substrate is fitted into the frame, the assembly is covered by a clean dry cover slip. Bubbles between the PDMS and glass are eliminated and the bond between the two materials promoted by carefully pressing on the assembly. The microfluidic capillary tubing (PEEK, internal diameter 360 μm; IDEX Health & Science LLC, Oak Harbor, WA, USA) is inserted gently into the ports and the system is ready to use. It easily allows flow-through rates of 2 mL/h, which is sufficient for reliable and fast continuous analysis. The waterproof and gas-tight seal was provided by a reversible bonding between PDMS and glass, which stems from van der Waals interactions [34]. Since the system is not permanently sealed (e.g., with oxygen plasma), it can withstand only a relatively limited pressure.

### 2.4. The Experimental Setup

**Figure 4:**
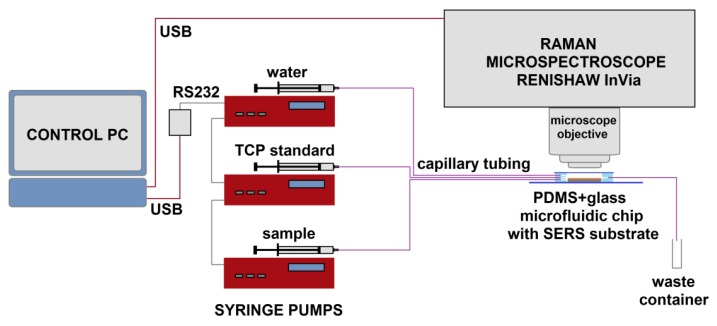
Schematic of the experimental setup for microfluidic SERS based quantitative detection of TCP (1,2,3-trichloropropane) and other chloroalkanes. The microfluidic chip with the SERS substrate is placed under the objective of the Raman microspectroscope and connected to the syringe pumps by capillary tubes. The syringe pumps and the spectroscope are controlled with the PC.

The experimental setup is schematized on Figure 4. We used an InVia spectrometer (Renishaw, Wotton-under-Edge, UK) for all the Raman spectroscopic measurements. The settings were following, unless specified otherwise: Laser power for Raman excitation was 24 mW in the sample plane at wavelength 785 nm. The integration time was 1s with 15 accumulations per spectrum, resulting in 15 s of acquisition time in total. We used 20×, 0.4 NA microscope objective (Leica, Wetzlar, Germany) for our measurements. For SERS, the excitation laser was focused on the substrate’s surface, while for conventional Raman (control measurements) the beam focus was adjusted for maximal signal inside a droplet of pure analyte.

The apparatus used for the sequential dilution of haloalkane TCP consisted of three microfluidic piston pumps (NE1001, New Era Pump Systems, Inc., Farmingdale, NY, USA) with manual and computer controlled regime (via USB to RS232 converter and LabView GUI), three 1 mL glass syringes (Hamilton, Bonaduz, Switzerland), luer-lock connectors (IDEX Health & Science LLC), and microfluidic tubing from the same manufacturer (PEEK, internal diameter 360 μm), which connected the chip to the syringes on one end (tubing length 35 cm each) and to a waste container on the opposite end (tubing length 15 cm). We used LabView-based software for controlling of the microfluidic pumps. The system is designed as a programmable syringe pump driver for 1–5 pumps. In all experiments, flow rates were kept at or below 2 mL/h.

### 2.5. Experimental Protocol

Deionized H_2_O was prepared in standard commercial purification system with resistivity about 5 MΩ/cm^2^. Sodium chloride, citric acid, disodium hydrogen phosphate and trichloromethane (CHCl_3_), all research grade purity, were supplied by Penta (Prague, Czech Republic). 1,2,3-Trichloropropane (TCP) and 2,3-dichloropropanol (DCP) of research grade purity were purchased from Sigma-Aldrich (St. Louis, MO, USA). Ten mM solutions of analytes were prepared by pipetting 10.6 µL, 9.6 µL and 8.0 µL of TCP, DCP and CHCl_3_ into 10 mL of deionized water, respectively. The analytes were solubilized by shaking the mixture for 2–3 min at room temperature in a glass flask. McIlvaine buffers of pH 4.0, pH 6.0, and pH 8.0 were made from prescribed volumes of 0.1 M citric acid solution and 0.2 M disodium hydrogen phosphate solution [35] and their pH was re-checked with pH-meter. The syringes were filled with solutions, bubbles were removed and the connectors plugged in. The ratio of the pumping speeds was used to dilute the solution in a stepwise manner. The resultant solution was pumped into the chamber with SERS substrate and measured by Raman spectroscopy.

### 2.6. Spectra and Data Processing

In order to extract quantitative information from the acquired spectra which contain fluorescence along with the Raman signal, we adopted the high-pass signal filter (Rolling Circle Filter —RCF) [36] to separate narrow Raman spectral peaks from the wide spectral background. With an appropriate choice of the filter parameters (filter width and number of filter passes) the background can be effectively removed with no significant distortion of the signal peaks. We kept the same filter parameters for all the measurements presented in this paper. Additionally, the spectra were de-noised by Savitzky-Golay filter. Spectral maps and various peak parameters were used for analysis of the obtained Raman spectra. The spectral processing and analysis were both realized via a homebuilt Raman analysis toolkit based on Matlab (MathWorks, Natick, MA, USA).

### 2.7. Gas Chromatography-Mass Spectroscopy

We used gas chromatography-mass spectroscopy (GC-MS) to test the retention of TCP in the microfluidic system. The configuration of the system was equivalent to that described in Section 2.4, except for using single pump. We measured and compared the input and output TCP concentrations of solutions prepared to contain 10 mM and 2 mM of TCP respectively. The flow rate of the TCP solution was 2 mL/h. Concentration of TCP was determined using a 7890 A gas chromatograph (Agilent, Santa Clara, CA, USA) equipped with ZB-5 column (30 m × 0.25 mm, film thickness 0.25 μm; Phenomenex, Torrance, CA, USA) and coupled with a 5975C MSD mass spectrometer (Agilent). The settings of the instruments were as follows: injector temperature 250 °C; carrier gas helium, constant flow rate 1.2 mL/min; oven temperature programmed from 40–150 °C at a rate of 20 °C/min; detector temperature 250 °C; quadrupole mass analyzer, electron impact, 70.3 eV; ion source temperature 230 °C; a.m.u. range 25–300. TCP retention time was 5.4 min.

## 3. Results

### 3.1. SERS Spectra of Tcp-Comparison of Sers Substrates SK307 with Klarite 312

**Figure 5:**
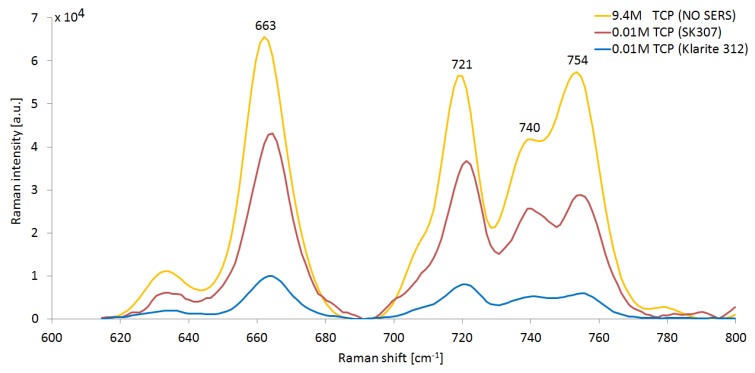
Comparison of conventional Raman spectrum of TCP with SERS on Klarite 312 and SK307. Conventional Raman spectrum was recorded from neat TCP, molar concentration 9.4 M. SERS spectra were recorded from 0.01 M (10 mM) TCP solutions on Klarite 312 and SK307. The horizontal axis represents Raman shift, while the vertical axis shows Raman (SERS) signal intensity. All the spectra were recorded with identical settings, as described in Section 2.4. The SERS peaks representing C-Cl stretching in TCP are present at 663 cm^−1^, 721 cm^−1^, 740 cm^−1^, and 754 cm^−1^. They are red shifted by about 1 cm^−1^ relative to the conventional Raman.

We observed SERS signals from C-Cl bonds in 10 mM TCP solution, collected on conventional Klarite 312 substrate and our homemade equivalent SK307. We compared these spectra with conventional Raman of neat TCP, see Figure 5. We measured the Raman (SERS) intensity of the dominant TCP peak at 663 cm^−1^. The SERS signal reached 1.0 × 10^4^ a.u. on Klarite 312 and 4.5 × 10^4^ a.u. on SK307. In comparison, the conventional Raman of neat TCP (molar concentration 9.4 M, 3 orders of magnitude higher than for SERS) yielded Raman intensity around 6.6 × 10^4^ a.u. under otherwise identical conditions. Conventional Raman spectroscopy of 10 mM TCP under these conditions showed no TCP signal. 

The signals of TCP are present at 663 cm^−1^, 721 cm^−1^, 740 cm^−1^, and 754 cm^−1^. All the peaks represent stretching vibrations of C-Cl bonds. The influence of molecular conformations of TCP and other analytes on the Raman shift are further discussed in Section 3.5.

We calculated the relative Raman signal amplification ***A*** for TCP by both SERS substrates Klarite 312 and SK307. Our definition of ***A*** is equivalent in all respects to the standing definition of the analytical enhancement factor (AEF) [10]. We defined I_663_ as the magnitude of the Raman signal of TCP at 663 cm^−1^ in a.u. Concentration of TCP c_TCP_ was given in moles per liter (mol/L; M) to adjust for different analyte concentrations. We calculated the corrected signal intensity: S = I_663_/c_TCP_ for both the SERS substrate (S_S_) and the conventional Raman (S_R_). Subsequently, we calculated the relative amplification ***A*** = S_S_/S_R_. These relations were defined assuming all optical components in the Raman microspectroscope and the excitation wavelength, laser power and integration time remained the same (see Section 2.4). While Klarite 312 afforded amplification ***A*** = 140, SK307 substrate reached ***A*** = 610 under the same conditions, giving about 4.3× better signal than Klarite. 

**Figure 6:**
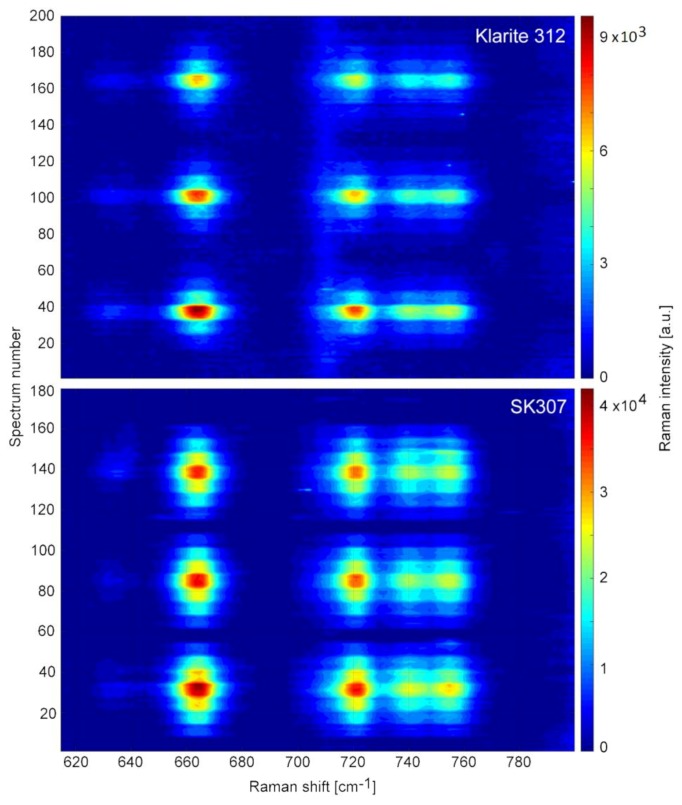
Spectral maps of TCP concentration gradients on SERS substrates Klarite 312 and SK307. TCP concentrations were 10.0, 7.5, 5.0, 2.5, and 0.0 mM. Vertical axis represents time ordered spectra (15 s/spectrum). Horizontal axis represents the Raman shift [cm^−1^]. The intensity of Raman signal is color-coded, see the side bar. The maps were created from spectra collected under identical conditions, except for a small difference in timing (50 versus 45 min total time). SK307 has stronger signal, more linear response to concentration changes and its signal loss is slower than with Klarite 312.

Although the results do not take in account the relation between Raman and SERS active volumes, they represent the relative increases of the analytically useful signal from the two examined SERS substrates compared to conventional Raman spectroscopy. We used spectral maps to visually aid the analysis of the TCP concentration gradients, see Figure 6.

Each spectral map shows three cycles of concentration step-gradients. Each spectrum was collected for 15 s, therefore the spectrum number relates to time in minutes in 4:1 ratio. We observed well defined steps of SERS signal change over the course of the experiment in both SERS substrates (Klarite 312 and SK307). On Klarite 312 the SERS signal intensity has gradually decreased over the course of three repetitions. Although with SK307 the tendency was similar, it was much less pronounced. We further investigated the magnitude of the SERS signal loss in both substrates over time and the overall linearity of the response to the TCP concentration changes. 

**Figure 7:**
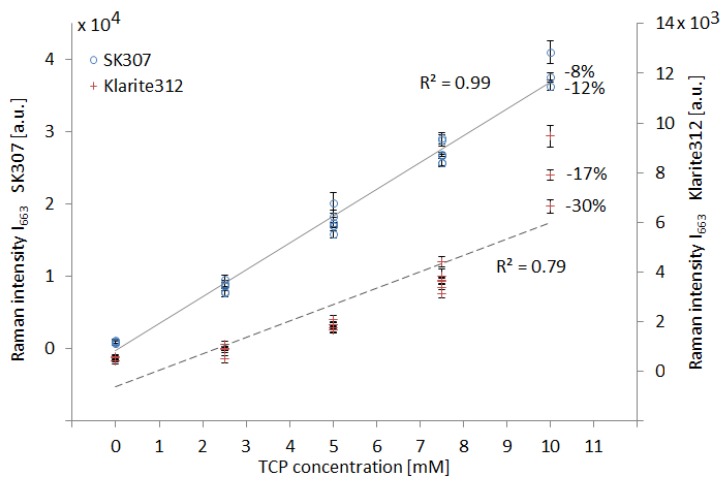
Comparison of TCP signal from SERS substrates Klarite 312 and SK307. Dependence of Raman signal intensity on TCP concentration. Raman intensity I_663_ represents the Raman signal magnitude at the indicated Raman shift. With Klarite 312, the response is not linear and the signal decays by 30% within 3 cycles. SK307 has nearly linear response and the signal decreases by only 12% within 3 cycles. This represents signal degradation 26% per hour. The linear regression (grey lines) yielded the regression coefficients R^2^. Their magnitude is proportional to the linearity of the data distribution. Each data point was averaged from 6 measurements. Error bars: 95% confidence interval.

The linearity of the SERS signal in response to increasing TCP concentration and the gradual loss of the SERS signal for both substrates are presented in graph on Figure 7. The Raman (SERS) intensity at 663 cm^−1^ (I_663_) is projected (separately for SK307 and Klarite 312) on the vertical axes, while the horizontal axis represents the TCP concentration. The concentration response curve of Klarite 312 to TCP was not found to be linear. Instead, the TCP signal appears to be close to the lower detection limit (LOD) in the lower range of concentrations (2.5–5.0 mM) as it becomes gradually too faint to detect. The observed signal loss over the course of the 3 cycles resulted on average in 30% reduction of the original signal intensity (with 10 mM TCP). The response of SK307 is very close to linear and the loss of the signal over the three cycles was only 12%. After comparing the general characteristics of SK307 with its “parent structure” Klarite 312, we continued by researching some other properties of the SK307 substrate.

### 3.2. Influence of Dissolved Salt on the TCP SERS Signal from SK307

**Figure 8:**
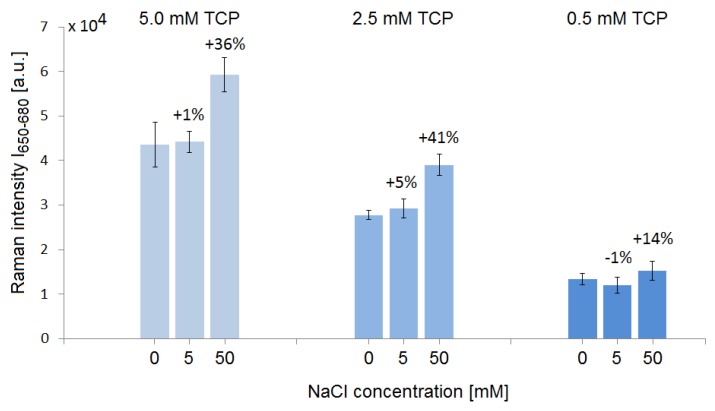
The effect of cross gradient of NaCl and TCP on SERS intensity with SK307 substrate. The horizontal axis represents the combinations of TCP and NaCl concentrations (in mM). Vertical axis shows the Raman intensity I_650–680_. The Raman intensity of the TCP signal increased significantly when 50 mM NaCl was present in the samples. Each data-point was averaged from 10 measurements. Error bars: 95% confidence interval.

We investigated the influence of dissolved salt on the SERS signal of TCP on the SK307 substrate, see Figure 8. To measure the Raman intensity, we numerically integrated the Raman peak with maximum at 663 cm^−1^ in an interval 650–680 cm^−1^, and we named this quantity I_650–680_. It was used because the signals of 0.5 mM TCP were weak and the integration over larger area offers significantly higher precision than single point measurement in such cases. We used NaCl solution and created a cross-gradient by mixing it automatically with the TCP solution and deionized water in the optofluidic chip to obtain the final concentrations of 0, 5, and 50 mM NaCl in 0.5, 2.5, and 5 mM TCP on the SERS substrate. We found that the addition of 50 mM NaCl increases the TCP SERS signal by up to 41% relative to the signal with no NaCl present. The effect may be due to the increased salt concentration reducing the solubility of TCP in water by increasing ionic strength, effectively pushing the TCP molecules out of the solution and maximizing the interaction of the molecules with the SERS substrate. We measured SERS spectra of further combinations of 5 mM TCP with NaCl in concentrations up to 1000 mM. We found that maximal signal was produced with 120 mM NaCl, adding up to 100% to the original signal intensity. 

### 3.3. Influence of pH on the TCP SERS Signal from SK307

**Figure 9:**
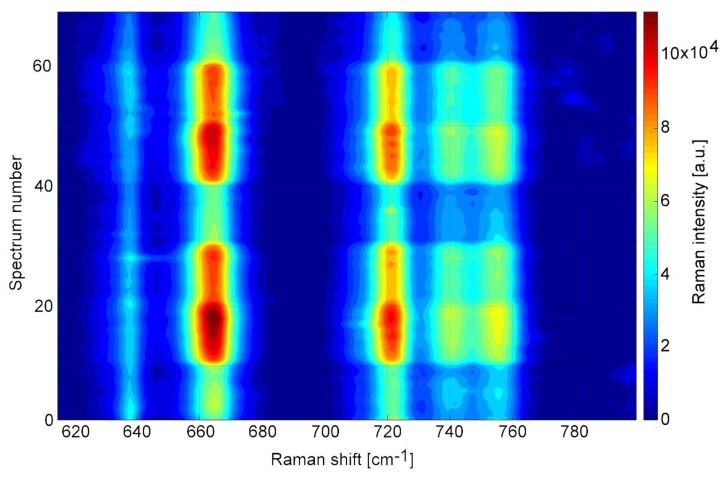
Spectral map of SK307 SERS signal of 10 mM TCP in cycling pH. Vertical axis represents time ordered spectra (30 s/spectrum). Horizontal axis represents the Raman shift. The intensity of the Raman signal is color-coded. Signal intensity is highest at pH 8.0 (red), decreases at pH 6.0 (orange) and is minimal at pH 4.0 (green-blue).

**Figure 10:**
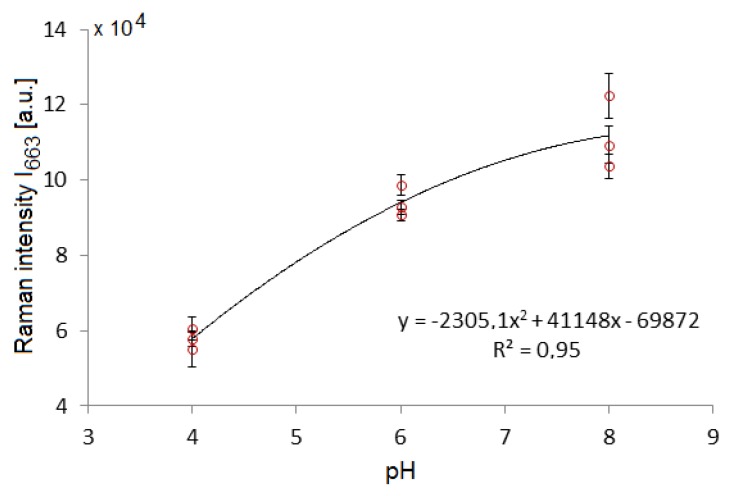
The pH dependence of the SERS signal from 10 mM TCP on SK307 substrate. We used data from 3 subsequent cycles of pH changes to plot the dependence. The regression curve approximates the relation between pH and the SERS signal. Each data point was averaged from 6 measurements. Error bars: 95% confidence interval.

10 mM TCP solutions in McIlvaine buffers of pH 4.0, 6.0 and 8.0 were prepared and sequentially injected into the microfluidic SERS chamber. The spectral map in Figure 9 represents a segment of the repeating sequence of pH induced changes in TCP spectra. All the observed TCP peaks follow a similar trend, whereby the signal intensity increases with higher pH. We measured intensities over 1.2 × 10^5^ at 663 cm^−1^ with pH 8.0 buffer containing 10 mM TCP, whereas in pH 4.0 the maximal intensity reaches only half that value, see Figure 10. Since the Raman signal of TCP showed tendency to increase with higher pH, we additionally tested the range up to pH 10.0 with borate buffers. We found the TCP signal continued to grow further with increasing pH, reaching 39% more TCP signal at pH 10.0 compared to pH 8.0, but the overall signal intensity over time decreased faster in pH > 8.0 (−50% of signal per hour) than in pH 8.0 or lower (−32% of signal per hour). Both these values compare negatively to the TCP signal decrease of 26% per hour reached without salt or buffer. There is clearly a tradeoff between the signal intensity and the signal stability. These phenomena are further discussed in Section 4.2.

Influence of pH on the SERS spectrum was observed previously with ionic species such as 4-mercaptobenzoic acid and exploited for pH sensing [37,38]. In such cases, the change in Raman spectra is linked to deprotonation of the analyte molecule and the intermolecular interactions, which can change how effectively the molecules pack on the substrate. In the case of TCP, deprotonation cannot take place, therefore these changes can be ascribed just to the intermolecular interactions between the TCP molecules and the ions in the solution. Similar ideas were already tested for improving the SERS detection of ionic species [39].

### 3.4. Measurement of Submillimolar Concentrations of TCP and a Simulated Sampling

**Figure 11:**
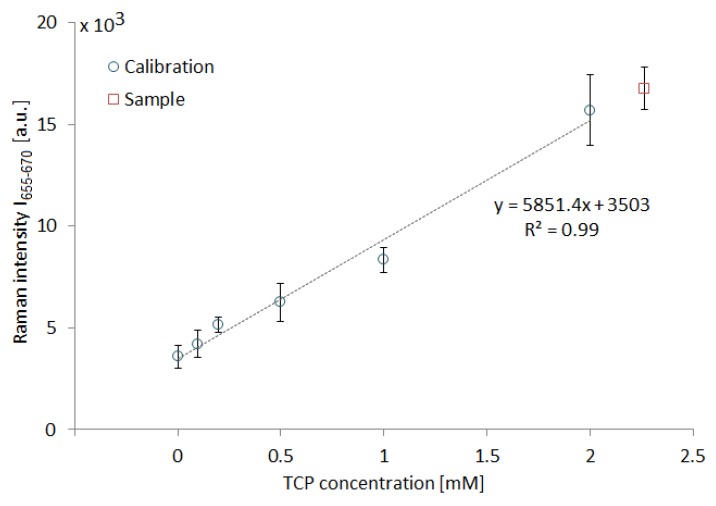
SERS of TCP on SK307 substrate: TCP calibration series (blue circles) and a separately injected sample (red square) of TCP solution (2 mM). Raman intensity I_655–670_ (vertical axis) represents numerical integral of the Raman intensity between the indicated Raman shifts. TCP concentration is shown on the horizontal axis. We were able to reliably detect 0.2 mM TCP in water. Calibration yields a linear dependence over the course of all calibration points even at concentrations reaching LOD. Each calibration data-point was averaged from 10 measurements. The data-point of the separately injected sample was averaged from 25 measurements. Error-bars: 95% confidence interval.

We attempted to calibrate the response of lower TCP concentrations on the SK307 substrate, see Figure 11. We used stepwise dilution of 2 mM TCP solution in distilled water down to 0.1 and 0 mM. After this calibration procedure, a simulated TCP sample in distilled water was automatically introduced from a separate syringe near the end of the process. This sample contained 2 mM TCP. In environmental monitoring application of the system, this would represent the actual sample of contaminated water. The integration time in this experiment was set to 30 s in order to maximize the collected SERS signal at lower TCP concentrations compared to the previous experiments. To measure the Raman intensity, we numerically integrated the Raman peak with maximum at 663 cm^−1^ in an interval 655–670 cm^−1^, (I_655–670_). Similarly to the NaCl experiment, it was used because the signals of TCP were weak and the integration over larger area offers higher signal to noise ratio and better accuracy than a single point measurement.

The resulting calibration series demonstrates the possibility to quantitatively measure submillimolar concentrations of TCP in water. While 0 mM TCP concentration was not statistically significantly different from 0.1 mM TCP, we were able to clearly distinguish 0.2 mM of TCP in the solution. This represents our LOD, equivalent to 30 ppm. The linear regression has shown that the SERS response of SK307 remains highly linear even in this range of concentrations, with the regression coefficient R^2^ = 0.99. The regression formula was used for calculation of the TCP concentration of the simulated sample. The calculated concentration of the sample was 2.27 mM (real concentration was 2.0 mM), 14% relative error of measurement. The sample was within the error margin of the 2 mM calibration standard. 

### 3.5. Other Analytes

**Figure 12:**
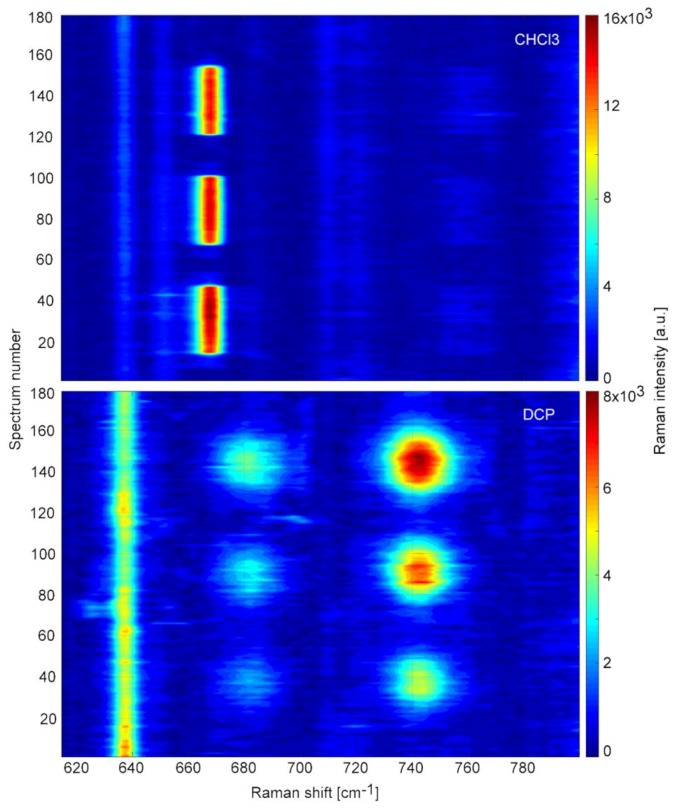
Spectral maps of 10 mM solutions of DCP (2,3-dichloropropanol) and CHCl_3_ (trichloromethane) on SK307 during 3× repeated step gradient from 0 to 10 mM and back to 0 with 2.5 mM steps. Vertical axis represents time ordered spectra (15 s/spectrum). Horizontal axis represents the Raman shift. The intensity of Raman signal is color-coded.

**Figure 13:**
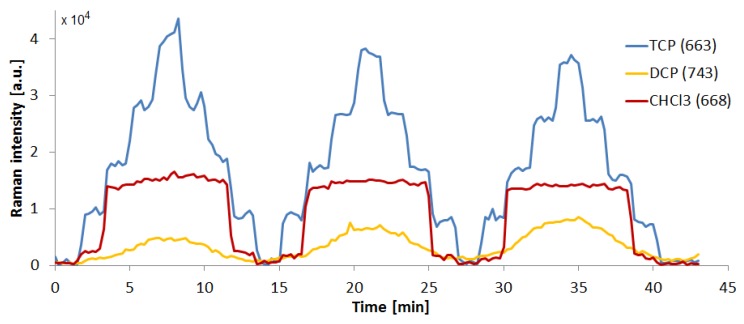
Time course of the SK307 SERS signal from TCP, DCP, and CHCl_3_ solutions during 3× repeated step gradient from 0 to 10 mM and back to 0 with 2.5 mM steps. Raman shift of the projected signal (in cm^−1^) is given in brackets along with the sample name. Note the various dynamics of the signal change. The pumping programs and spectral acquisition parameters were identical in all three samples.

We experimented with SK307 substrate in combination with different analytes than TCP, such as metabolites of enzymatic degradation pathways, namely, 10 mM solutions of 2,3-dichloropropanol (DCP), epichlorohydrin (ECH) and chloropropane-2,3-diol (CDP) in deionized water. Additionally, we analyzed 10 mM solution of trichloromethane (CHCl_3_), a frequently used laboratory solvent with properties similar to TCP. All measurement settings were identical to the previous experiment with TCP. The signal of 10 mM ECH and CDP solutions was undetectable, probably due to the low proportion of only one Cl atom per molecule and their relatively hydrophilic nature. We have successfully detected CHCl_3_ and DCP. The spectral maps on Figure 12 present three repetitions of a step-gradient similar to the one for TCP. Here as well the solutions used contained 10, 7.5, 5.0, 2.5, and 0 mM of the analyte, nevertheless, in neither case the map reminds the one of TCP and in both cases the stepwise nature of the gradient is no longer obvious. The cross sectional view of the spectral maps of TCP, DCP and CHCl_3_ through the maxima of the most prominent peaks is depicted on Figure 13. 

2.5 mM CHCl_3_ resulted in barely discernible SERS signal, which from 5 mM upwards became abruptly significant, but almost did not increase towards the highest concentrations. The signal fell again when the concentration reached 2.5 mM. This on-off behavior may point out to an early saturation of the substrate with the CHCl_3_ molecules, but the true cause is still unclear. The lowest measured concentration of 2.5 mM CHCl_3_ is equivalent to 300 ppm, but the SERS signal was strong enough to easily allow detection of 100 ppm CHCl_3_. 

**Figure 14:**
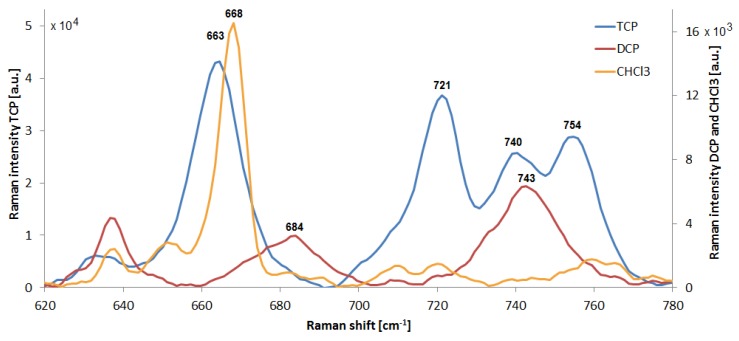
SK307 SERS spectra of 10 mM solutions of TCP, DCP, and CHCl_3_. Horizontal axis represents Raman shift, while the vertical axes show Raman (SERS) signal intensity separately for TCP (**Left**) and for DCP, and CHCl_3_ (**Right**). All the annotated peaks represent C-Cl bond stretching. The differences in Raman shift are caused by alternative conformations of the atoms in the respective molecule.

The SERS signal of DCP appeared to roughly follow the concentration, albeit the stepwise nature of the gradient is not readily apparent. This behavior may be linked to the more polar character of the analyte, which is caused by the hydroxyl group on the first carbon. This polar part may be allowing stronger attachment to the SERS substrate. We repeatedly observed that the SERS signal of DCP becomes gradually stronger with increasing number of testing cycles. This is unexpected, given that so far we observed with all analytes that the signal decays over time. The reasons for this are so far unclear. The hydroxyl group may hypothetically serve to react with photo-oxidized impurities and therefore to revive the Au nanostructures. The lowest measured concentration of 2.5 mM DCP is equivalent to 320 ppm, but the SERS signal was strong enough to easily allow detection of 100 ppm DCP. In conditions such as presented in Section 3.4, the LOD for both DCP and CHCl_3_ could be reduced even more. The SK307 SERS spectra of DCP, CHCl_3_ and TCP are presented in Figure 14. All the peaks represent stretching vibrations of C-Cl bonds.

The Raman shift differences are caused by the various surroundings of the C-Cl bond in the molecule. In TCP, distribution of several staggered conformers gives rise to the four main TCP peaks in this spectral window. According to the literature [40], there are six such conformers, but only some of them are energetically favorable: 663 cm^−1^ vibration of TCP is C-Cl stretch G+G- combined with 662 cm^−1^ C-Cl str. AG-, 719 (721) cm^−1^ is C-Cl str. AG-, 742 (740) cm^−1^ C-Cl str. AG+, and 753 (754) cm^−1^ C-Cl str. AG-. “A” stands for “anti” conformer, while “G” refers to “gauche”. In comparison with TCP, DCP appears to have only two preferred ways to arrange its C-Cl bonds, presumably the G conformer vibrates at 684 cm^−1^ and A at 743 cm^−1^. DCP SERS signal reached maximal intensity around 1 × 10^4^ a.u., at 742 cm^−1^, with 15 × 1 s accumulations and 10 mM DCP concentration. Chloroform has only one possible conformation of C-Cl bond, with peak signal at 668 cm^−1^. Its SERS signal reached maximal intensity around 1.6 × 10^3^ a.u. at 668 cm^−1^, with 15 × 1 s accumulations and 10 mM CHCl_3_ concentration.

### 3.6. Retention of TCP in the Microfluidic System

We assessed the retention of TCP in the microfluidic system containing the SK307 SERS substrate under the defined working conditions. We realized this by comparing the concentrations of TCP in the solutions flowing in and out of the system, respectively, measured by GC-MS. There was a loss of TCP in the 2 mM and 10 mM TCP solutions before entering the microfluidic system, TCP concentrations being reduced by 6% to 1.88 mM and 15% to 8.49 mM, respectively. TCP concentrations on the output of the chip were 1.67 mM and 5.82 mM, respectively. The retention of TCP in the microfluidic system was 11% for 2 mM and 32% for 10 mM TCP solution. These results suggest that the retention decreases with lower TCP concentrations, with the total concentration difference −42% for 10 mM TCP , but only below −17% for the measurements of TCP concentration 2 mM or less. The losses can be mainly ascribed to the volatility of TCP, and its nonpolar character which allows it to pass through PDMS. Glass and gold are non-permeable to TCP, however, they may adsorb some TCP on the surface. We did not correct our results with respect to these findings.

## 4. Discussion

### 4.1. Using the Microfluidic SERS Detector with SK307 for Environmental Monitoring

TCP is a persistent organic pollutant and probable human carcinogen. It does not contaminate soil, but it readily leaks into the groundwater. One of the proposed strategies for decontamination of water from TCP is bioremediation. Haloalkane dehalogenase was engineered for enhanced efficiency of the degradation of TCP [41] and coupled with another two enzymes haloalcohol dehalogenase and epoxide hydrolase to convert TCP via several intermediates to glycerol and chloride ions [30,31,32,42]. The bacteria containing these enzymes are tested to be used for bioremediation purposes [41,43]. In order to measure the rate of the enzymatic degradation of TCP, the SERS detector based on SK307 may be employed. The idea of reaction monitoring in situ by SERS has been widely realized [44,45]. The success of this approach would depend on the rate of fouling of the SERS substrate by the enzyme and its degradation products. The sensitivity of SK307 SERS substrate is so far not sufficient to allow for effective direct measurement of water samples contaminated with less than about 200 µM (30 ppm) TCP. However, if this method was coupled with pre-concentration of the analyte, such measurement would be possible. Pre-concentration coupled with SERS was previously described [23]. The LOD could be further decreased by changes of the ratio of the pyramid sides [46], or possibly by using more sophisticated structures such as dimer on mirror [14], or highly porous 3-D Au coated nanostructures [47,48]. Our SERS substrate was fabricated on a silicon platform using electron beam lithography, which is not suitable for mass production, but ideal for prototyping. In our system, the reusability of the Si part of the substrate and the microfluidic part allows for very economical and ecological workflow. For mass production, all the necessary structures used in our experiment could be easily fabricated e.g., by nanoimprint lithography and transferred to pressure molded housings. 

We experimented with several different types of SERS substrates, both suspended nano-colloids, such as spheres, core-shell particles, nano-urchins, nano-rods, etc. and with nanostructured surfaces such as various nano-pillars, nano-fishnets, and nano-pyramids. We have found that, among all the other substrates that we tested, the Raman signal of (TCP) and other chlorinated alkanes was enhanced only by the structure consisting of inverted nano-pyramids etched into silicon monocrystalline plate, covered by a gold layer of defined thickness and roughness, such as Klarite 312 and SK307. The improved SERS signal of SK307 compared to Klarite 312 was probably caused by the decreased depth of the pit [33] and by the increased number of the inverted pyramids in the area illuminated by the laser. The thickness and granulation of the Au layer was very similar to Klarite, probably playing only a minor role in the observed difference. To our best knowledge, there are only a few publications dealing with SERS on simple aliphatic chlorinated hydrocarbons [49,50]. In the latter publication, the authors describe detection of CHCl_3_ using electro-generated SERS substrate (ESERS). They were able to detect about 100 ppm of CHCl_3_ in a water solution of 100 mM KBr.

### 4.2. Gradual Loss of the SERS Signal over Time

Laser light used for SERS excitation on SK307 may be responsible for the loss of the SERS signal. We propose a few possible mechanisms of the laser-induced substrate degradation. A direct mechanism can be linked to the heating of the substrate with a focused laser beam. The composite structure of the substrate makes it vulnerable to the thermally induced delamination of the Au layers from the silicone base. The Au film deforms and loses the optimal shape in the process, leading to the overall decrease in SERS efficiency. An indirect mechanism can be linked to the photoinduced chemical fouling of the SERS substrate, in which case the laser catalyzes the chemical entities present in the solution to oxidize and/or polymerize on the SERS substrate. Additionally, the laser could hasten deposition of nano- to micrometer sized floating debris onto the substrate simply by its radiation pressure. We have observed inverse tendency of gradual signal increase from the SERS substrate over time with DCP. If this proved in the future to be a phenomenon linked with some chemical changes of the substrate, it may be useful for boosting the signal of other molecules. We acknowledge that the gradual loss of the SERS signal from SK307 complicates the analysis over longer time span. However, this can be remediated by short but frequent calibrations. With a single calibration on the start, with SK307 one can measure samples for full 20 min with error from the accuracy drop below 10%. We observed increased signal loss with NaCl and buffers as well. The mechanisms may include decrease in surface energy and subsequent enhanced intercalation of the solution between the gold and the silicon below, accessible through imperfections in the gold layer. Different mechanism may involve increased deposition of nonpolar impurities from the solution of high ionic strength.
